# Gap Junction Protein Beta 2 Gene Variants and Non-Syndromic Hearing Impairment among Couples Referred For Prenatal Diagnosis in the Northeast of Iran

**Published:** 2019-03

**Authors:** Samaneh Vojdani, Reza Jafarzadeh Esfehani, Vahid Iranmanesh, Hafezeh Davari, Nafiseh Amini, Mohammad Ehsan Jaripour, Peyman Zargari, Mahtab Dastpak, Ariane Sadrnabavi

**Affiliations:** 1 *Department of Medical Genetics, School of Medicine, Mashhad University of Medical Sciences, Mashhad, Iran. *; 2 *Medical Genetic Research Center, School of Medicine, Mashhad University of Medical Sciences, Mashhad, Iran. *; 3 *Department of Genetic, Academic Center for Education, Culture and Research (ACECR)-Khorasan Razavi, Mashhad, Iran. *

**Keywords:** Hearing loss, Gap Junction Protein Beta 2, Genetic variant

## Abstract

**Introduction:**

Hearing impairment is a complex medical disorder which has genetic and non-genetic causes. Gap Junction Protein Beta 2 (GJB2) gene variant is a well-known disease-causing gene among patients with hearing impairment. The frequencies of genetic variants in the GJB2 gene are different in each population. This study aimed to discuss the GJB2 gene status in an Iranian population with hearing impairment who referred for prenatal testing.

**Materials and Methods:**

This cross-sectional study was conducted in a genetic laboratory affiliated with Mashhad Jahad Daneshgahi, Mashhad, Iran. A total number of 21 bilateral hearing impaired patients were enrolled in this study. The exons for target GJB2 gene were amplified by polymerase chain reaction after the confirmation of the hearing impairment and the exclusion of the acquired causes of hearing loss.

**Results:**

The c.35delG and c.79G>A variants were the first and second most common variants in the study population, respectively. The mean age of the patients was 27.5 (8.7) years and 12 cases were male. There was no significant association between hearing impairment degree and age and heterozygosity status (P=0.376 and P=.074 respectively).

**Conclusion:**

The c.35delG and c.79G>A variants were determined as the first and second most common variants in the GJB2 gene, respectively. The mean age of 26 years in this study population indicates the late referral for the evaluation of the hearing difficulty. Furthermore, it highlights the further need to encourage families with a history of hearing impairment to engage in genetic counseling.

## Introduction

The incidence of hearing impairment is about one out of 166 newborns in Iran in which it is much more prevalent than the rest of the world (1). Hearing loss is a complex disorder which can be classified according to its etiology, type, and age. Genetic causes of hearing loss are responsible for approximately more than half of hearing impaired cases (2). 

Syndromic and non-syndromic hearing impairment is a classification, which mostly relies on the genetic basis of the disorder. Syndromic patients show other clinical abnormalities in addition to hearing problems, whereas hearing impairment is only observed among non-syndromic patients (2).

Gap junction-related genes, Ion channel-related genes, and tight junction-related genes are 3 well-known genes which are responsible for most cases of non-syndromic hearing loss (1). 

The GJB2 gene, which is a variant of gap junction-related genes, is responsible for the hearing impairment in Iran and many other countries. 

It has been reported that GJB2 gene variants will result in prelingual hearing loss (2). Different regions of the world show diversities among different GJB2 gene variants, which are also prominent in Iran (3,4). Although there are several screening methods and carrier detection techniques for the diagnosis of non-syndromic hearing impairment, this clinical condition is still a great burden to those who are affected (5). 

Incomplete population coverage of detection techniques, poor screening tests, and hearing impairment denial of both parents and physicians are reported to be the three main reasons for late diagnosis and the management of patients with hearing loss (6).

Therefore, it is necessary to determine the late referral cases and establish cost-effective genetic testing for hearing loss according to each country’s prevalent variants. Accordingly, it may lead to achieve a successful strategy to reduce the burden of this disorder (5). 

The present study was conducted in Iran in order to achieve this goal and also evaluate the genetic diagnostic age for hearing impairment among patients with autosomal recessive non-syndromic hearing loss. 

## Materials and Methods

This cross-sectional study was conducted in a genetic laboratory affiliated with Mashhad Jahad Daneshgahi, Mashhad , Iran, and was approved by Khorasan Academic Centre for Education, Culture and Research. In total, 21 hearing impaired patients were selected out of 200 individuals referring for prenatal diagnosis between August 2015 and January 2018. With regard to the ethnicity, all the participants were Persian and resident in Mashhad. 

The hearing impairment was confirmed by an otorhinolaryngologist and required clinical tests, such as pure-tone audiometry through a diagnostic audiometer.

Furthermore, imaging studies were performed where indicated. Pure-tone averages of more than 25 decibels hearing level (dBHL) were defined as hearing loss (mean dBHL at 500, 1000, and 2000 Hz).The severity of deafness was divided into mild (24-40 dB), moderate (41-55 dB), moderately severe (71-90 dB), severe (71-90 dB), and deep (90 dB). Those couples who had acquired hearing loss because of environmental causes (e.g., infection, noise, drug ototoxicity, and trauma) were excluded from the study. The informed consent and 5ml of venous blood sample were obtained from all participants. 

Genomic DNA was extracted from peripheral blood by salting out method. Individual exons for target GJB2 gene were amplified by polymerase chain reaction (PCR). Oligonu- cleotide primer pairs for the amplification of each exon were designed online by Primer 3. 

The PCR was conducted in the 20μL final volume and was performed in the Labcycler (SensoQuest, Germany) under the following conditions: 94 °C for 5 min followed by 35 cycles of 94 °C for 30 sec, 60 °C for 30 sec, and 72 °C for 30 sec. As a final extension, 72 °C was applied for 5 min (Table.1). 

Moreover, amplified fragments were separated on 1.5% agarose gel electrophoresis. All PCR products were sequenced (MPI, Germany) and they were analyzed using Chromas software (Version 2.4.4).

The sequence alignment was performed by reference sequences from ENSEMBLE (ENSG00000165474) and the sequence databases for GJB2 gene. The data were analyzed in SPSS software (version 20) through Kruskal Wallis and the Chi-square tests. 

**Table1:** Polymerase chain reaction program

**Final Extension**	**Extension**	**Annealing**	**Denaturation**	**Pre Denaturation**	**Stage**
Temperature	95˚C	95˚C	60˚C	72˚C	72˚C
Duration	5 min	30 sec	30 sec	30 sec	10 min
Cycles	1	35			1

## Results

A total number of 21 patients referred for prenatal diagnosis was enrolled in this cross-sectional study. The mean age of patients was 27.5 (8.7) years and 12 cases were male. According to the results obtained from the Kruskal Wallis test, no significant associations were observed regarding the study variables. There were not any significant associations between the hearing impairment degree and the age and heterozygosity status (P=0.376 and P=.074 respectively). Table 2 demonstrates the results of the variant analysis. The c.35delG variant was the first most common variant identified in this study. Three subjects with profound deafness were homozygous for this variant. However, one patient with profound hearing loss exhibited compound heterozygous (c.341A>G). The second most common variant was c.79G>A. Three subjects with profound deafness were heterozygote and one of the cases was compound heterozygote (c.341A>G). The third variant was c.380G>A, which was found in two heterozygote patients with profound and moderate deafness. 

**Fig 1:**
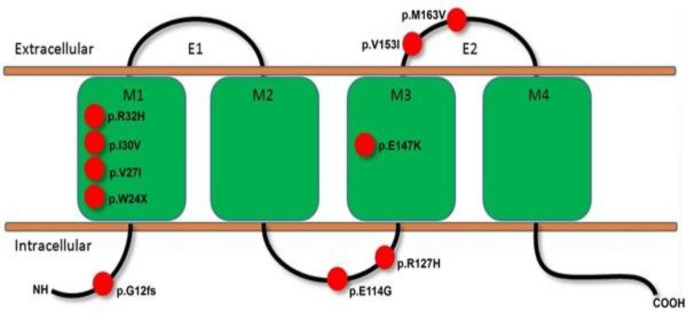
Predicting topology of the connexin 26 protein (cx26). The cx26 protein contains four transmembrane domains (M1–M4), two extracellular domains (EC1, EC2) and intracellular (N- and C-terminal) domains. The red circle indicating the location of GJB2 mutations which identified among patients with non-syndromic hearing loss

Heterozygote variant of c.487A>G was found in two participants with profound and moderate deafness. Moreover, homozygote variants were identified in one of the healthy participants. Three cases with profound hearing loss had c.457G>A, c.95G>A, and c.88A>G. The results showed that individuals without hearing loss had c.487A>G variant in GJB2 gene. Furthermore, most of the variants were located in the M1 domain of connexin 26 protein (Fig.1). 

**Table 2:** Frequencies of discovered GJB2 variants

**N**	**Transcript consequent**	**rs number**	**Protein consequent**	**Variant type**	**Number of patients**	Allele frequency Iranome*	**Allele frequency** ENSEMBL**	**Allel frequency Exac**	**Functional effect**
1	c.487A>G	rs80338949	p.Met163Val	Missense	2	0.001250	0.9996006389776	0.0001652	Pathogenic
2	c.457G>A	rs111033186	p.Val153Ile	Missense	2	0.03125	0.013	0.01057	Pathogenic
3	c.380G>A	rs111033196	p.Arg127His	Missense	2	0.01063	0.0003993610223	0.01537	Benign
4	c.341A>G	rs2274083	p.Glu114Gly	Missense	2	0.01000	0.969	0.01459	Benign
5	c.439G>A	NA	p. Glu147Lys	Missense	1	-	0.969	8.261e	Pathogenic
6	c.95G>A	rs111033190	p.Arg32His	Missense	2	0.0006250	0.99995922166	8.24e-06	Pathogenic
7	c.79G>A	rs2274084	p.Val27Ile	Missense	4	0.01937	0.07	5.767e-05	Benign
8	c.88A>G	rs374625633	p.Ile30val	Missense	1	-	0.9999632929	0.04538	Pathogenic
9	c.71G>A	rs104894396	p.W24*	Frameshift	1	-	0.0003993610223	0.0005767	Pathogenic
10	c.35delG	rs80338939	p.Gly12fs	Frameshift	6	0.001875	0.99997140527	0.00604	Pathogenic

* www.iranome.ir

** www.ensemble.org

## Discussion

Approximately, 40% of Iranian population has the genetic background for autosomal recessive non-syndromic hearing loss (7). This fact has made the hearing impairment as a complex disease with a great burden (5). The hearing impairment caused by GJB2 variant is not fully correlated with patient’s phenotype and shows incomplete penetrance. Modifier genes and environmental factors are two main causes for this phenotype-genotype miscorrelation. It is necessary to determine the modifier genes in order to achieve better phenotype prediction (8). According to the results, 35delG and c*.*79G*>*A were the first and second most common variants among study participants, respectively. Although frameshift variant of 35delG is responsible for 20% of hearing loss in Caucasians, some countries (e.g., Greece) have reported high prevalence rates. This variant is responsible for one-third of prelingual and sensorineural hearing loss in Greece population (9). In a multicenter study conducted by Snoeckx et al. on African and Asian population, c*.*35delG and* p.*V37I were reported as the most common mutant alleles among Arab (65%) and Ashkenazi Jewish patients (37%), respectively. 

Although there is no significant association between GJB2 variants and hearing loss degree, it has been reported that truncating variants are associated with a greater degree of hearing loss in contrast to non-truncating variants (8). Regardless of the genetic challenges of hearing loss, mild to moderate hearing impairment has always been a big concern for both clinician and patients. 

Many parents are unaware of their children's hearing impairment in their early life. A recent study conducted in Iran has shown that approximately one-third of parents are unaware of their children hearing loss (10). Parents may deny the fact that their children response to vocal stimuli irregularly and has a hearing impairment. Robertson et al. called this denial as “wishful thinking” which will result in delayed diagnosis of hearing problems and even make the children recovery poorer (6). Therefore, it is mandatory to educate the general population which results in the reduction of the number of hearing-impaired patients and increase of public awareness of prenatal testing for hearing loss. In contrast to developing educating programs, paying attention to diagnostic programs also seems noteworthy. Some types of deafness cannot be diagnosed with postnatal hearing tests while these tests have low sensitivity and accuracy among progressive and late-onset cases (11).

 A ten-year cohort study has demonstrated the value of hearing screening at birth. Moreover, deafness has been diagnosed in one-quarter of the children at school. Such findings demonstrate the obvious necessity of postneonatal care pathways for the identification of the missed ones. Otherwise, these children will face various difficulties in their later life (12). This issue highlights the role of genetic testing in the early diagnosis of hearing impairment. As a result, gene detection techniques have become a corner stone in detecting the causes of hearing loss (11). However, in Iran, many people are still not aware of genetic counseling for many diseases, such as hearing problems. The mean age of couples referring for prenatal testing for GJB2 variant in this study was 26 years. None of these patients with non-syndromic hearing loss were genetically tested or had premarital genetic counseling. A possible reason for this delay in diagnosis could be the cultural issues.

Traditional intermarriage among deaf people is a common finding in some countries which will increase the number of deaf people in some situations. The genetic testing for GJB2 variant among partners who wish to marry each other and has hearing impairment will predict the possibility of having a hearing impaired child if other genetic and non-genetic causes of hearing loss can be ruled out. However, this mate selection may be forbidden in some cultures and may raise ethical issues (13). Full awareness of the most common genetic variations in the population leads to the establishment of appropriate genetic tests for prenatal screening which is cost-effective and efficient. 

## Conclusion

It is necessary to screen all children with hearing impairment to determine the specific gene variants regardless of the hearing impairment degree. Therefore, further programs for family planning and conducting prenatal diagnosis become easier following the determination of these variants in each population. The mean age of 26 years in this study population indicates the late referral for the evaluation of the hearing difficulty. Moreover, it highlights the further need for public education regarding genetic counseling among families with a history of hearing impairment. Furthermore, a more close relationship between clinicians and geneticists will facilitate further experimental procedures for these families. 
